# Characterization and Chemical Management of *Phytophthora palmivora* and *Fusarium incarnatum-equiseti* Species Complex Associated with Durian Root Rot in China

**DOI:** 10.3390/microorganisms14092061

**Published:** 2026-09-15

**Authors:** Yun Xing, Houya Fang, Yijia Deng, Changsheng Lu, Taorui Wu, Ting Wei, Shilong Chen, Hongna Wang, Zhiwen Wang, Xili Liu

**Affiliations:** 1 Sanya Institute, China Agricultural University, Sanya 572000, China; xy13115609818@126.com (Y.X.); fanghouya@cau.edu.cn (H.F.);; 2 State Key Laboratory of Agricultural and Forestry Biosecurity, Ministry of Agriculture and Rural Affairs (MARA), Key Laboratory of Pest Monitoring and Green Management, College of Plant Protection, China Agricultural University, Beijing 100193, China; 3 State Key Laboratory of Agricultural and Forestry Biosecurity, Key Laboratory of Biopesticide and Chemical Biology, Ministry of Education, College of Plant Protection, Fujian Agriculture and Forestry University, Fuzhou 350002, China; 4 State Key Laboratory of Crop Stress Resistance and High-Efficiency Production, College of Plant Protection, Northwest A&F University, Yangling 712100, China

**Keywords:** soilborne disease, oomycete, fungicide sensitivity, co-inoculation, synergistic mixture

## Abstract

Durian cultivation has become a key development industry in China in recent years, and durian root rot (DRR) constitutes a major threat to production. In this study, symptomatic root and stem base tissues and leaves were systematically collected from the main durian-producing regions of China, yielding 202 fungal and oomycete isolates. Isolates were identified based on morphological characterization and multi-locus phylogenetic analysis. *Fusarium* spp. and *Phytophthora* spp. were the most frequently recovered taxa, accounting for 39.11% and 16.34% of the isolates, respectively. Koch’s postulates confirmed *Phytophthora palmivora* and the *Fusarium incarnatum-equiseti* species complex (FIESC) as the causal agents. In controlled co-inoculation experiments, simultaneous inoculation with both pathogens resulted in a shorter latent period and increased disease severity compared with single inoculations, indicating a potential interaction that warrants further investigation. Fungicide sensitivity assays showed that *P. palmivora* was sensitive to oxathiapiprolin, mandipropamid, fluopicolide, and chlorothalonil, while metalaxyl-M, mancozeb, cymoxanil, and propamocarb hydrochloride showed poor inhibitory activity. FIESC exhibited sensitivity to tebuconazole, fludioxonil, and pydiflumetofen but was insensitive to difenoconazole, mefentrifluconazole, and phenamacril. Oxathiapiprolin and azoxystrobin mixed at ratios of 1:13.8 and 1:10.5 produced pronounced synergistic effects against *P. palmivora*, with synergy ratios of 3.57 and 2.91 and co-toxicity coefficients of 357.98 and 291.08. This study compared three application methods (root drenching, trunk painting, and trunk injection) of fungicides and identified root drenching as the most effective strategy. Subsequent pairwise screening of eight combinations, composed of four anti-oomycete fungicides (oxathiapiprolin, mandipropamid, fluopicolide, azoxystrobin) paired with two anti-fungal fungicides (tebuconazole, fludioxonil), was conducted via 0.2 L root drenching. Among them, the tebuconazole-azoxystrobin combination offered the highest control efficacy (98.95%), whereas tebuconazole-mandipropamid exhibited the lowest. Further ratio optimization of the tebuconazole-azoxystrobin mixture, performed using a reduced application volume of 0.1 L per plant, identified a 1:2.36 ratio as the most effective formulation, providing 94.65% control. Overall, this work clarifies the pathogen complex associated with DRR in China, provides preliminary evidence for enhanced disease severity during co-inoculation of *P*. *palmivora* and FIESC, and identifies optimized fungicide combinations and application methods under greenhouse conditions. These results provide a foundation for further field validation and for the development of integrated management strategies.

## 1. Introduction

Durian (*Durio zibethinus*) is a seasonal tropical fruit prized for its soft, creamy flesh and rich flavor [1]. It likewise serves as an abundant provider of essential trace elements, minerals, and vitamins. Studies have indicated that durian possesses anti-diabetic and lipid-lowering properties and may help reduce the risk of cardiovascular disease and inflammation [2]. Durian is widely acclaimed across the globe, particularly by Chinese buyers; China’s dominance in global durian imports has spurred enhancements throughout the durian processing and product chain. The major durian-producing countries are Thailand, Vietnam, and Malaysia, which together cultivate nearly 250,000 hectares [3]. Over the past few years, the cultivation of durian has grown rapidly in China and has become a very promising tropical agricultural sector. However, the expansion has been accompanied by the movement of pathogen-infected planting materials across regions, facilitating the introduction and early establishment of pathogens in new planting areas. Coupled with multi-season accumulation of soilborne inoculum, durian root rot (DRR) has become the most serious threat across the major durian-growing regions. The disease typically causes stunted growth, leaf yellowing, wilting, and even whole-plant death [4,5,6]. Previous studies have shown that a range of pathogens can infect durian roots, including *Phytophthora palmivora*, *Phytophthora capsici*, and *Fusarium* spp. [7]. *P. palmivora* is recognized as a highly destructive pathogen and the most important causal agent of durian root rot (DRR), which can result in complete tree mortality. *P. palmivora* is capable of infecting various durian tissues, causing root rot, stem canker, leaf blight, dieback of seedlings and mature trees, and pre- and post-harvest fruit rot [8,9,10]. Moreover, it infects a wide range of other tropical crops, including cocoa [11], oil palm [12], rubber [13], coconut [14], and cassava [10], inducing diverse symptoms in different plant parts. This broad host range allows the pathogen to cycle among alternate hosts and persist across growing seasons [15], posing a particularly severe threat to durian production. Remarkably, *P. palmivora* has already inflicted heavy economic losses: in the Davao region of Mindanao, Philippines, disease incidence can reach 40%, potentially resulting in approximately 25% tree mortality [16]. Although the pathogens associated with DRR have been identified in some well-studied durian-growing regions, they remain poorly characterized in many other cultivation areas, especially in China, where durian production has expanded rapidly. This gap in pathogen identification strongly impedes the development of targeted and effective disease management strategies.

*P. palmivora* belongs to oomycetes, which are phylogenetically distinct organisms with cellulose-based cell walls and a fundamentally different repertoire of molecular targets, resulting in a far more limited arsenal of effective anti-oomycete molecules [17,18]. It primarily relies on motile zoospores that sense host-derived chemical signals in soil and water to locate roots [19,20]. Upon infecting the root system, the pathogen penetrates the plant cell wall through a combination of mechanical force and enzymatic degradation [21], ultimately compromising vascular function. In addition to oomycetes, *Fusarium* spp. represent another important group of pathogens responsible for vascular wilts and root rots in numerous crops. These two groups of pathogens not only vary in their infection mechanisms but also necessitate different fungicide types for effective control, thus complicating disease management when they appear together in co-infections. In durian, only certain *Fusarium* spp. are capable of causing diseases independently. For instance, foliar diseases are associated with *F*. *pernambucanum*, *F*. *sulawesiense*, *F*. *hainanense*, *F*. *irregulare*, *F*. *mangiferae* and *F*. *concentricum*, several of which belong to the *F. fujikuroi* species complex (FFSC) [22]. Furthermore *F*. *incarnatum* and *F*. *solani*, members of the *Fusarium incarnatum*-*equiseti* species complex (FIESC) and the *F. solani* species complex (FSSC), respectively, have been reported to cause DRR in southern Thailand [23]. Additionally, *Fusarium* spp. can establish co-infection complexes with oomycete pathogens such as *P*. *capsici* and *P*. *sojae*. A synergistic interaction is described between these two groups of pathogens. For example, during co-infection with *P*. *sojae*, *Fusarium* spp. can produce vitamin B6, which facilitates the infection process of the oomycete [24]. In tobacco, co-infection by *Fusarium* spp. and *P*. *nicotianae* leads to imbalanced rhizosphere soil physicochemical properties, negatively affecting plant growth [25]. Such interspecific synergy, compounded by the paucity of effective anti-oomycete chemistries, renders disease management considerably more difficult. To date, the precise pathogenic makeup and fungicide susceptibility profiles linked to DRR in China have not been systematically documented. This knowledge gap leaves precise disease management without the necessary theoretical foundation and poses a serious challenge to the effective control of the disease.

Chemical control remains the primary approach for managing soilborne pathogens due to its rapid action and ease of application. In durian-growing regions of Southeast Asia, commonly used fungicides against *P*. *palmivora* include metalaxyl, mefenoxam, mancozeb, and fosetyl-Al [26]. However, prolonged and repeated use of a single active ingredient readily selects for resistant pathogen populations [27]. For instance, *P*. *palmivora* isolates from southern Thailand exhibited high and progressively increasing levels of resistance to metalaxyl [28]. In northern Thailand, *Fusarium* spp. recovered from muskmelon fruit rot displayed reduced sensitivity to carbendazim [29], while azoxystrobin-resistant *F. weifangense* has been documented on Indian jujube (*Ziziphus mauritiana*) [30]. The emergence of resistance severely compromises disease control efficacy [31]. Rational combinations of fungicides with distinct modes of action not only broaden the target spectrum and improve control but also exploit complementarity among different biochemical targets to achieve synergistic effects and delay the onset of resistance [32,33].

Root and stem base diseases caused by *P*. *palmivora* and *Fusarium* spp. are particularly difficult to manage because the primary infection site is situated at the root–stem junction near the soil surface. Sprays targeting the leaves, like those used on durian, often fail to effectively reach the infected root–stem area, as the dense canopy interferes with droplet interception and gravity prevents the solution from moving downward, severely limiting deposition [34]. Consequently, screening and optimizing targeted application methods—such as trunk injection, root drenching, and trunk painting—is essential to deliver the active ingredient precisely to the infection site, thus enhancing both pesticide use efficiency and overall disease control [35].

Essentially, identifying the pathogen composition and implementing a logical chemical management strategy are necessary for managing the present production issues. To this end, this study aimed to provide a basis for the control of DRR in China by addressing the following objectives: (1) identifying the causal agents responsible for DRR; (2) evaluating the sensitivity of the dominant pathogens to commonly used fungicides; (3) screening for fungicide combinations with high efficacy against both pathogens; and (4) determining the optimal field application method for the candidate mixtures. The co-inoculation response described in this study should be regarded as a preliminary observation requiring further quantitative evaluation under field conditions. The findings will not only offer critical technical support for the sustainable development of the durian industry in China but also provide a framework for the management of DRR in other durian-growing regions.

## 2. Materials and Methods

Unless otherwise stated, general chemicals and media components were purchased from Coolaber, Beijing, China. Technical-grade fungicide sources and SHAM details are provided in Section 2.6 and Supplementary Table S2.

### 2.1. Collection, Isolation and Preservation of DRR Pathogen

Durian root and stem base samples showing typical root rot symptoms were collected from the main durian-growing areas in Hainan Province, China, during the years 2024 and 2025. Putative pathogens were isolated using the tissue isolation method [36] and the leaf disc baiting method [37]. Tissue isolation: diseased tissue pieces from the advancing margin of lesions were washed free of surface soil and debris, cut into small segments, surface-disinfected with 75% ethanol for 30 s, rinsed twice with sterile distilled water, and placed onto two types of selective media: (i) V8 juice agar (V8A) amended with a selective agent cocktail (ampicillin, rifampicin, carbendazim, and pentachloronitrobenzene, each at 50 μg/mL final concentration) to suppress bacterial and non-target fungal growth; and (ii) potato dextrose agar (PDA) amended with 100 μg/mL ampicillin. Plates were incubated at 28 °C in the dark. Leaf disc baiting: ten grams of soil from the rhizosphere of diseased plants was suspended in 10 mL of sterile water containing the same selective agent cocktail at the above concentrations. Fresh, surface-disinfected healthy durian leaf pieces (1 cm^2^) were added, and the mixture was incubated under alternating light and dark cycles for 3 days. The leaf pieces were then transferred to the two selective media described above. Following colony development, isolates were then purified through hyphal-tip transfer. Purified isolates were maintained on V8A and PDA slants at 15 °C. For morphological characterization, isolates were subcultured onto carrot agar (CA) and PDA and incubated at 28 °C for 3 days, after which the surface and reverse colony morphology were recorded. Microscopic features were examined under a light microscope (Nikon ECLIPSE Ci Series, Beijing, China).

### 2.2. DNA Extraction, PCR Amplification and Sequencing

Suspected pathogens were inoculated onto V8A and PDA plates overlaid with cellophane membranes and incubated at 28 °C for 3 days. Mycelia were harvested, and total genomic DNA was extracted using the CTAB method [38]. DNA concentration was determined with a microvolume spectrophotometer (Thermo Fisher Scientific Inc., Beijing, China), and integrity was assessed by electrophoresis on 1% agarose gels. Different genetic markers were targeted for PCR amplification according to the pathogen genus. For *Fusarium* isolates, the internal transcribed spacer (ITS), translation elongation factor 1-α (*EF-1α*), and RNA polymerase II second largest subunit (*rpb2*) were amplified; for *Phytophthora* isolates, ITS, *EF-1α*, and *β-tubulin* were amplified. Primer sequences are provided in Supplementary Table S1. Each 50 μL PCR mixture contained 25 μL of 2 × Taq MasterMix (Accurate Biotechnology, Changsha, Hunan, China), 1 μL of each forward and reverse primer (10 μM), 200 ng of genomic DNA, and distilled water to 50 μL. The thermal cycling program consisted of an initial denaturation at 94 °C for 3 min, followed by 35 cycles of 94 °C for 30 s, 57 °C–60 °C for 30 s, and 72 °C for 60 s, with a final extension at 72 °C for 10 min. PCR products were verified by 1% agarose gel electrophoresis and subsequently subjected to bidirectional Sanger sequencing at Beijing Qingke Biotechnology (Beijing, China).

### 2.3. Sequence Analysis and Phylogenetic Tree Construction

To avoid bias arising from the relatively large sample size, representative isolates from diverse collection sites and host plants are used to cover the major clades revealed by the multilocus phylogeny. This strategy ensures that the subsequent sensitivity and efficacy data will not be dominated by a single genotype or geographic origin.

Raw sequences were assembled using SnapGene 6.0.2. Reference sequences for *Fusarium* spp. and *Phytophthora* spp. were downloaded from the NCBI database (https://www.ncbi.nlm.nih.gov/). Each locus was aligned separately using MAFFT v7.490 with the auto option and subsequently trimmed using trimAl v1.4 under the automated1 mode. The multi-locus datasets were then concatenated into a single supermatrix. The best-fit substitution model for the concatenated dataset was determined by ModelFinder in IQ-TREE v2.2.0 based on the Bayesian Information Criterion (BIC). Maximum Likelihood (ML) trees were inferred using IQ-TREE with 1000 ultrafast bootstrap replicates. Bayesian Inference (BI) was performed in MrBayes v3.2.7 with two independent runs of four MCMC chains for 2,000,000 generations, sampling every 100 generations and discarding the first 25% as burn-in. Nodal support is indicated by ML bootstrap values ≥70% and Bayesian posterior probabilities ≥0.95. *Phytopythium* spp. and *Alternaria alternata* were used as outgroups for *Phytophthora* spp. and *Fusarium* spp., respectively. To root the phylogenetic trees, outgroup taxa were selected based on their phylogenetic proximity to the ingroups: *A. alternata* was used as the outgroup for the *Fusarium* dataset, representing the closest sister lineage within Sordariomycetes, while *Phytopythium* spp. were used for the *Phytophthora* dataset as the immediate sister clade within *Peronosporales*.

### 2.4. Pathogenicity Tests

To assess the pathogenicity of the isolates and fulfill Koch’s postulates, in preliminary tests for Koch’s postulates verification, leaves were more susceptible to infection and symptom development than roots and stem bases. Isolates that caused symptoms on leaves did not necessarily infect the root/stem base of durian, whereas almost all isolates capable of infecting the root/stem base also caused symptoms on leaves. Given that root inoculation is time-consuming and costly, detached leaves were selected as the initial screening material for Koch’s postulates verification, to improve screening efficiency and reduce costs. Healthy 6-month-old ‘Monthong’ and ‘Musang King’ cuttings (purchased from Hainan Liuxiangyuan Agricultural Technology Development Co., Ltd., Ledong, Hainan, China) were inoculated using three methods: spore suspension root drenching, detached leaf inoculation, and stem inoculation. *Phytophthora* spp. and *Fusarium* spp. were induced to sporulate separately, as follows: *Phytophthora* spp. was cultured on CA medium (200 g of carrots were homogenized and filtered. The solution was adjusted to a final volume of 1 L and supplemented with 1.5% agar) at 28 °C under alternating light and darkness; *Fusarium* spp. was cultured in mung bean liquid medium with shaking at 150 rpm at 28 °C. After sporulation, CA plates with *Phytophthora* spp. colonies were flooded with 10 mL of sterile distilled water, chilled at 4 °C for 45 min, and then kept at room temperature for another 45 min to stimulate zoospore release. The liquid cultures of *Fusarium* spp. were filtered through two layers of sterile gauze to harvest conidial suspensions. Spore concentrations were determined with a hemocytometer and adjusted to 1 × 10^6^ spores/mL with sterile distilled water. All spore suspensions were prepared immediately before inoculation. The three inoculation methods were carried out as follows. Root drenching: spore suspension (1 × 10^6^ spores/mL, 30 mL per seedling) was applied to the stem base. Detached leaf inoculation: a through-and-through puncture wound was made on the leaf surface using a sterile needle probe 1 mm in diameter, and 10 μL droplets of spore suspension (1 × 10^6^ spores/mL) were deposited onto each wound. Stem inoculation: a single puncture wound approximately 1 cm deep was created on the stem surface using the same sterile 1.5 mm needle probe, and a 5 mm mycelial plug from an actively growing culture was inserted; the site was then covered with moistened sterile cotton and wrapped with plastic film to maintain high humidity. For all treatments and controls, wounds of the same size, depth, and number (one per leaf inoculation site or one per seedling) were made using the same tool and procedure. All inoculated plants and detached tissues were maintained in a greenhouse or growth chamber at 28 °C and 80% relative humidity and were monitored regularly for symptom development. The development of symptoms was observed until obvious symptoms appeared, and then photos were taken. Once typical symptoms appeared, the pathogen was re-isolated from the diseased tissues using the tissue isolation method and then compared with the original inoculum morphologically and molecularly.

### 2.5. Disease Severity Assessment and Data Calculation

For the root drenching assay, disease severity was evaluated using a modified scale based on Burpee (1980) and supporting references [39,40]. The original 0–10 disease rating scale of Burpee (1980) [39] was adapted for DRR by retaining six non-continuous odd grades (0, 1, 3, 5, 7, and 9), each corresponding to a typical disease stage from asymptomatic to plant death. The use of odd grades helps minimize the tendency of raters to choose intermediate values, thereby improving the reliability and reproducibility of disease assessment. The six grades are: 0, no symptoms; 1, mild yellowing of one or two leaves; 3, obvious yellowing/wilting with localized brown lesions at the collar; 5, extensive yellowing/defoliation with expanded collar lesions; 7, complete defoliation with root constriction; and 9, plant wilting with necrotic roots and a severely constricted, desiccated collar. For the stem inoculation assay, severity was rated on a five-grade scale according to the percentage of lesion coverage on the stem surface: 0, no lesion; 1, ≤5%; 2, 6–25%; 3, 26–50%; and 4, ≥50%.

The disease index (DI) was calculated following Abdelaziz et al. [41]:

(1) $$\mathrm{DI}=\frac{\sum \left(\mathrm{Number}\;\mathrm{of}\;\mathrm{plants}\;\mathrm{per grade}\times \mathrm{Gradescore}\right)\times 100}{\mathrm{Total}\;\mathrm{number}\;\mathrm{of}\;\mathrm{plants}\times \mathrm{Maximum}\;\mathrm{gradescore}}$$

Disease severity on detached leaves was expressed as relative lesion area (RLA), calculated following Wang et al. [42]:

(2) $$\mathrm{RLA}(\%)=\frac{\mathrm{Total}\;\mathrm{lesion}\;\mathrm{area}}{\mathrm{Total}\;\mathrm{leaf}\;\mathrm{area}}\times 100$$

The extent of lesion expansion on inoculated stems was quantified as relative lesion length (RLL), adapted from Huang et al. [43]:

(3) $$\mathrm{RLL}(\%)=\frac{\mathrm{Lesion}\;\mathrm{length}}{\mathrm{Total}\;\mathrm{inoculated}\;\mathrm{stem}\;\mathrm{length}}\times 100$$

### 2.6. In Vitro Fungicide Sensitivity Assays

The in vitro sensitivity of the isolates was tested against 20 fungicides commonly used in agricultural production: metalaxyl-M, azoxystrobin (azo), pyraclostrobin, dimethomorph, chlorothalonil, mancozeb, fluopicolide, mandipropamid, fluopimomide, cyazofamid, fluazinam, cymoxanil, oxathiapiprolin (oxa), propamocarb hydrochloride, tebuconazole (teb), phenamacril, difenoconazole, pydiflumetofen, mefentrifluconazole, and fludioxonil. The mycelial growth rate method was employed to assess sensitivity.

A series of stock solutions of each technical-grade fungicide were prepared in dimethyl sulfoxide (DMSO) at different concentrations. Each stock solution was then diluted 1:1000 into autoclaved PDA cooled to approximately 50 °C to achieve the desired final concentrations shown in Table S2. The media were mixed thoroughly and dispensed into Petri dishes. For the sensitivity assay of azoxystrobin, salicylhydroxamic acid (SHAM) was additionally added to the cooled PDA at a final concentration of 50 μg/mL (50 ppm) to inhibit alternative oxidase-mediated respiration. Mycelial plugs (5 mm in diameter) cut from the actively growing margin of 3-day-old cultures were transferred to the center of the fungicide-amended plates. After incubation at 28 °C in the dark for 3 days, colony diameters were measured using the perpendicular intersection method. Each treatment included three replicate plates.

### 2.7. Preparation of Fungicide Mixtures and Sensitivity Testing

Oxathiapiprolin and azoxystrobin were combined at five mass ratios: 1:13.8, 1:10.5, 1:8, 1:5.5 and 1:3.3. These ratios were determined based on the registered field-use concentration ranges on the China Pesticide Information Network (http://www.chinapesticide.org.cn/): oxathiapiprolin is registered at 26–50 ppm and azoxystrobin at 165–360 ppm. Accordingly, the maximum mass ratio was calculated as 26:360 = 1:13.8, and the minimum as 50:165 = 1:3.3; five ratios spanning this interval were selected. For each ratio, the concentration gradient was derived from the single-agent sensitivity data, and concentrations were chosen so that the combined additive efficacy of the two active ingredients fell within the EC_10_-EC_90_ range. Technical-grade materials of each fungicide were weighed individually according to the designated ratios, dissolved in DMSO, and serially diluted as described in Section 2.6. The preparation of fungicide-amended plates and the measurement of mycelial growth inhibition followed the protocols detailed in Section 2.6.

The median effective concentration (EC_50_) of each mixture was estimated from the log-probit regression of concentration-response data. Synergistic ratios (SRs) were calculated using the Wadley method [44]:

(4) $${EC}_{50(theoretical)}=\frac{1}{\frac{Fraction\;of\;oxa}{{EC}_{50(oxa)}}+\frac{Fraction\;of\;azo}{{EC}_{50(azo)}}}$$

(5) $$\mathrm{SR}=\frac{{\mathrm{EC}}_{50(\mathrm{theoretical})}}{{\mathrm{EC}}_{50(\mathrm{observed})}}$$

SR > 1.5 was interpreted as synergism, 0.5 < SR < 1.5 as additivity, and SR < 0.5 as antagonism.

The co-toxicity coefficient (CTC) method of Sun and Johnson (1960) [45] was applied in parallel to evaluate the combined activity of the mixtures. Oxathiapiprolin was designated as the reference fungicide, and the following parameters were calculated:

(6) $$\mathrm{Toxicity}\;\mathrm{index}\;(\mathrm{TI})=\frac{{\mathrm{EC}}_{50}\;\mathrm{of}\;\mathrm{the}\;\mathrm{reference}\;\mathrm{fungicide}}{{\mathrm{EC}}_{50}\;\mathrm{of}\;\mathrm{the}\;\mathrm{tested}\;\mathrm{fungicide}}$$

(7) $$\mathrm{Actualtoxicity}\;\mathrm{index}\;(\mathrm{ATI})=\frac{{\mathrm{EC}}_{50\;}\mathrm{of}\;\mathrm{oxa}}{{\mathrm{EC}}_{50\;}\mathrm{of}\;\mathrm{themixture}}$$

(8) $$\mathrm{Theoretical}\;\mathrm{toxicity}\;\mathrm{index}\;(\mathrm{TTI})={\mathrm{TI}}_{\mathrm{oxa}}\times {\mathrm{Fraction}}_{\mathrm{oxa}}+{\;\mathrm{TI}}_{\mathrm{azo}}\times {\mathrm{Fraction}}_{\mathrm{azo}}$$

(9) $$\mathrm{CTC}=\frac{\mathrm{ATI}}{\mathrm{TTI}}\times 100$$

CTC > 120 was considered synergistic, 80 ≤ CTC ≤ 120 as additive, and CTC < 80 as antagonistic.

### 2.8. Greenhouse Efficacy Evaluation

Healthy, uniformly grown 1-year-old durian plants were used for greenhouse efficacy trials. The optimal fungicide mixture selected from in vitro screening was assessed for protective effects on both detached leaves and whole plants. For detached leaves, uniformly sized leaves were excised and placed in a moist chamber, sprayed evenly with the fungicide mixture, and inoculated 24 h later with 10 μL of a spore suspension (1 × 10^6^ spores/mL) of *Phytophthora* spp. alone (for efficacy evaluation of oomycete fungicide combinations) or with 10 μL of a mixed spore suspension (1 × 10^6^ spores/mL, 1:1) of *Phytophthora* spp. and *Fusarium* spp. (for efficacy evaluation of combinations of fungal and oomycete fungicides). Inoculated leaves were incubated at 28 °C under high humidity for 3 days, after which lesion development was recorded.

For whole plants, three application methods were compared: root drench (0.2 L or 0.1 L per plant applied to the stem base), trunk injection (0.05 L per plant slowly injected using a 50 mL syringe at multiple sites around the trunk circumference), and trunk painting (the mixture, formulated with a polyethylene glycol film-forming agent, was brushed onto the entire trunk circumference from 1 cm to 30 cm above the soil line, using 50 mL per plant). Distilled water served as the control.

For the greenhouse efficacy tests involving combinations of four oomycete fungicides and two fungal fungicides, the mixing ratios were determined based on the maximum field-use concentrations listed by the China Pesticide Information Network (http://www.chinapesticide.org.cn/). For the tebuconazole-azoxystrobin combination, the ratios were set using the maximum and minimum registered application concentrations: tebuconazole at 100–120 ppm and azoxystrobin at 140–395 ppm for fungal diseases, giving the highest ratio of 1:3.95, the lowest ratio of 1:1.16, and an intermediate ratio of 1:2.36.

All fungicide treatments were prepared from commercial formulations. The concentrations are provided in Supplementary Table S3. All working solutions were used immediately after preparation. At 24 h post-treatment, each plant was inoculated with 200 mL of a mixed spore suspension (1 × 10^6^ spores/mL, 1:1) of *Phytophthora* spp. and *Fusarium* spp. Each treatment included three plants with two replicates. Disease index was assessed on day 20 after inoculation to evaluate control efficacy.

### 2.9. Data Analysis

All experiments were performed in three independent replicates. Statistical analyses were conducted using SPSS v27.0 (IBM Corp., Armonk, NY, USA). Significant differences among treatment means were determined by one-way analysis of variance (ANOVA) followed by Duncan’s multiple range test. Graphs were generated with GraphPad Prism v9.5 (GraphPad Software, San Diego, CA, USA). A significance level of *p* < 0.05 was adopted for all comparisons.

## 3. Results

### 3.1. Field Symptoms and Isolate Diversity of DRR

**Figure 1:**
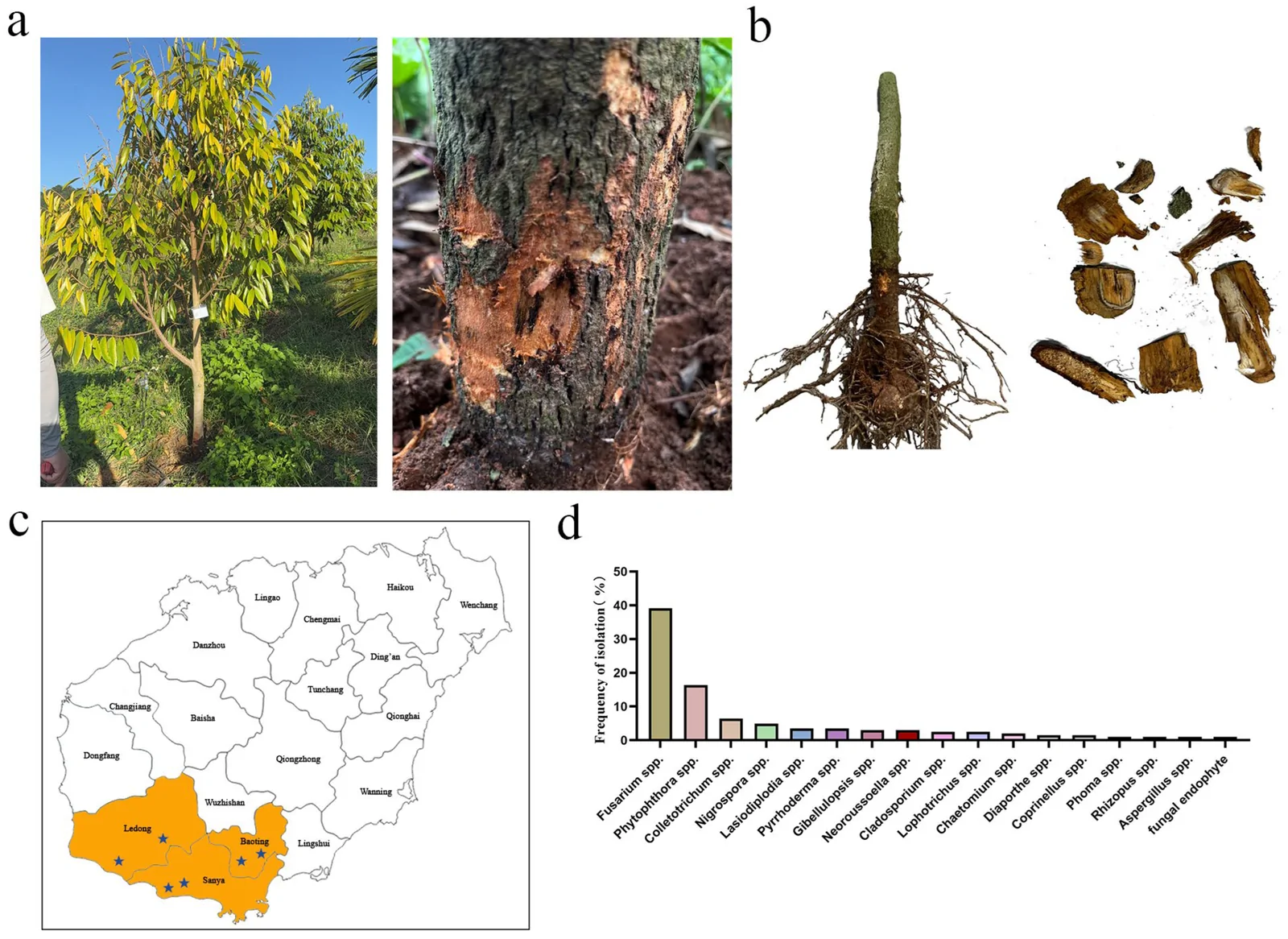
Field symptoms, geographic distribution, and pathogen community composition of DRR in China. (**a**) Aboveground symptoms of an affected durian tree, showing pronounced leaf yellowing and canopy decline. (**b**) Belowground and collar symptoms: (**Left**): an excavated root system; (**right**): a cross-section of infected root/collar tissue revealing extensive cortical decay and xylem browning. (**c**) Distribution of major durian-growing areas and sampling sites in Hainan Province, China. Orange-shaded regions indicate the principal durian production zones, and asterisks denote the locations where diseased samples were collected in this study. The sampling sites include Yipo New Village and Jiusuo Town in Ledong County, Dazuiniao Durian Base in Tianya District and Dazuiniao Durian Base in the Yucai Ecological Zone in Sanya City, and Sandao Farm and Shimucun Road in Baoting County. (**d**) Relative isolation frequencies (%) of the dominant fungal isolates recovered from symptomatic tissues, based on morphological and molecular identification.

Systematic surveys of DRR were conducted across Hainan Province, China, from April 2024 to March 2025. Symptomatic trees displayed marked decline, characterized by sparse foliage and leaf yellowing (Figure 1a); severely affected plants exhibited defoliation, branch dieback, root decay and constriction, and vascular discoloration—symptoms typical of root rot (Figure 1b). Twenty-one diseased samples were collected from Sanya, Baoting, and Ledong (Figure 1c). To maximize the recovery of putative pathogens, both tissue isolation and leaf disc baiting were performed, yielding 202 isolates in total. Preliminary ITS-based identification indicated that *Fusarium* spp. predominated (39.11%), followed by *Phytophthora* spp. (16.34%) (Figure 1d). Among them, 13 samples simultaneously yielded both *Phytophthora* spp. and *Fusarium* spp.

**Figure 2:**
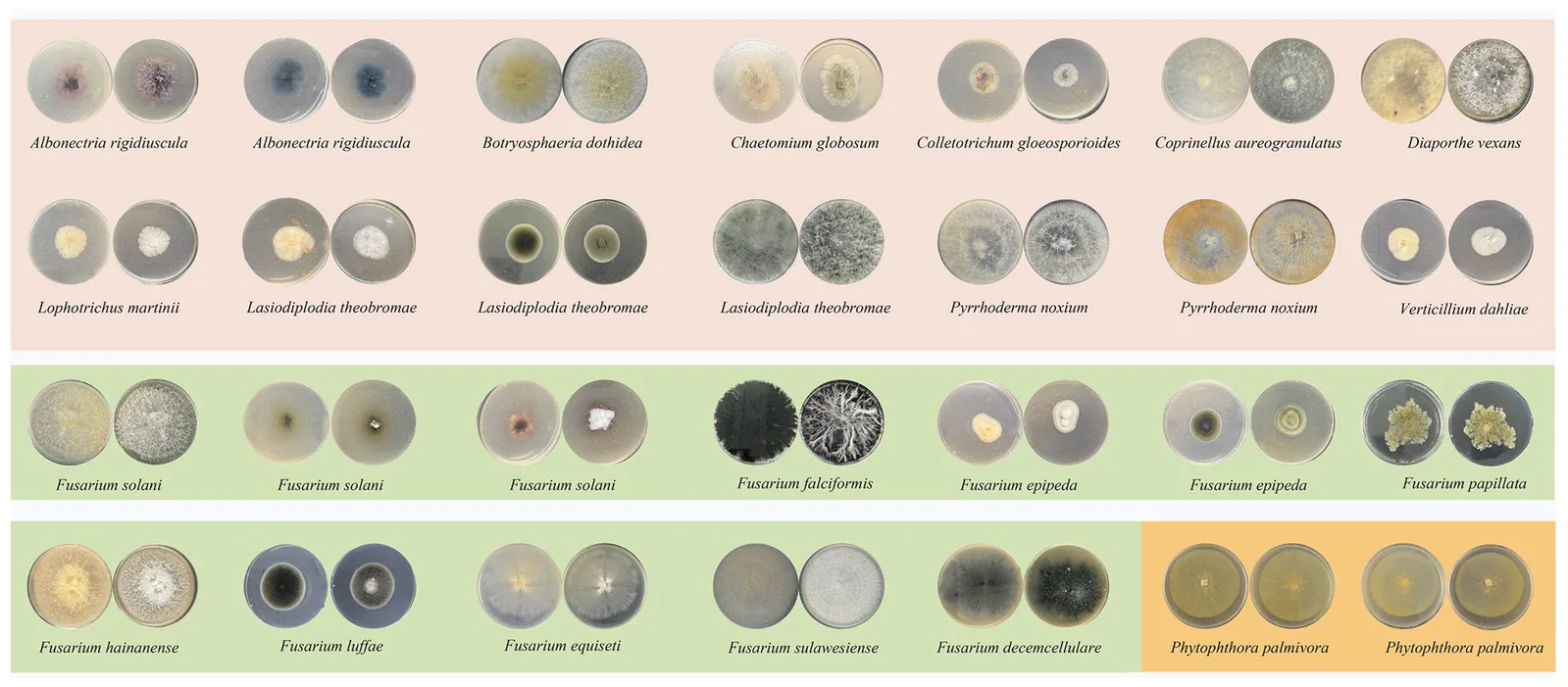
Colony morphology of representative isolates associated with DRR. Colony morphology of representative isolates of *P. palmivora* and *F. incarnatum*-*equiseti* species complex on PDA after 7 d of incubation at 28 °C, showing upper (**top**) and reverse (**bottom**) surfaces.

Given the taxonomic complexity of *Fusarium* spp. and the large number of isolates recovered, the *Fusarium* spp. isolates were initially grouped into 12 clusters based on colony morphology and ITS sequences (Figure 2). A preliminary fulfillment of Koch’s postulates, conducted by inoculating detached leaves, revealed that only isolates from the Fum-3 cluster were pathogenic. Accordingly, all three isolates comprising this cluster (designated FIEHN 16–18) were subjected to detailed characterization. For *P. palmivora*, four representative isolates (PPHN 1–4) were selected based on their distinct geographic origins and recovery from different diseased plants, ensuring that the selected isolates covered the geographic and host-source diversity of the pathogen population.

Morphologically, isolates PPHN 1–4 produced uniformly radial mycelia on CA, with sparse to moderate aerial hyphae that penetrated the medium. Sporangia were relatively small (32.5–45 µm), predominantly ellipsoidal to obpyriform, and variable in shape and size (Figure S1); chlamydospores were globose. Each sporangium released 18–51 reniform zoospores (10–13 µm). The optimal temperature for mycelial growth ranged from 24 to 28 °C. Isolates FIESC 16–18 formed yellowish-white colonies on PDA with a yellow reverse. FIEHN-17 grew more slowly than FIEHN-16 and FIEHN-18, and the colony margins of FIEHN-16 and FIEHN-17 were irregular. Conidia were falcate to fusiform, measuring 21.7–42.1 µm; hyphae were brownish or hyaline (Figure S1).

**Figure 3:**
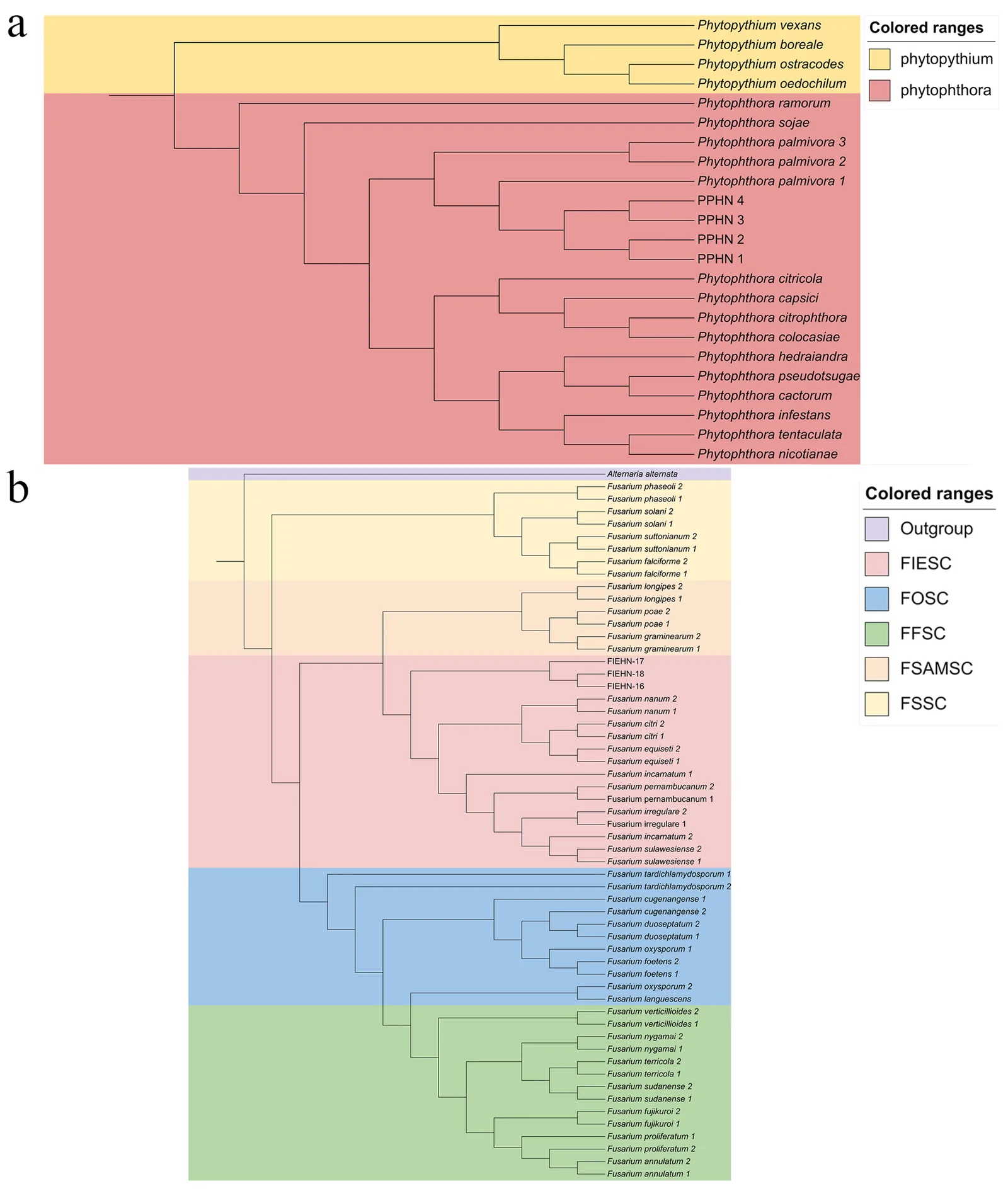
Multi-locus phylogenetic analysis of isolates associated with durian root rot (DRR). (**a**) Phylogenetic tree of *Phytophthora* spp. reconstructed from combined ITS, *β-tubulin*, and *EF-1α* sequences. (**b**) Phylogenetic tree of *Fusarium* spp. reconstructed from combined ITS, *EF-1α*, and *rpb2* sequences. The trees were inferred using Maximum Likelihood (ML) and Bayesian Inference (BI).

Multi-locus phylogenetic analysis of concatenated ITS, *β-tubulin*, and *EF-1α* sequences placed all four *Phytophthora* spp. isolates (PPHN 1–4) in a highly supported clade together with *P*. *palmivora* reference isolates (Figure 3a), corroborating the morphological identification. For *Fusarium*, analysis of concatenated ITS, *EF-1α*, and *rpb2* sequences resolved the three FIEHN 16–18 isolates within the *Fusarium incarnatum*-*equiseti* species complex (FIESC) (Figure 3b). Collectively, the morphological and molecular data confirmed that the dominant pathogenic agents were *P*. *palmivora* and FIESC.

### 3.2. Pathogenicity Assessment and Fulfillment of Koch’s Postulates

**Figure 4:**
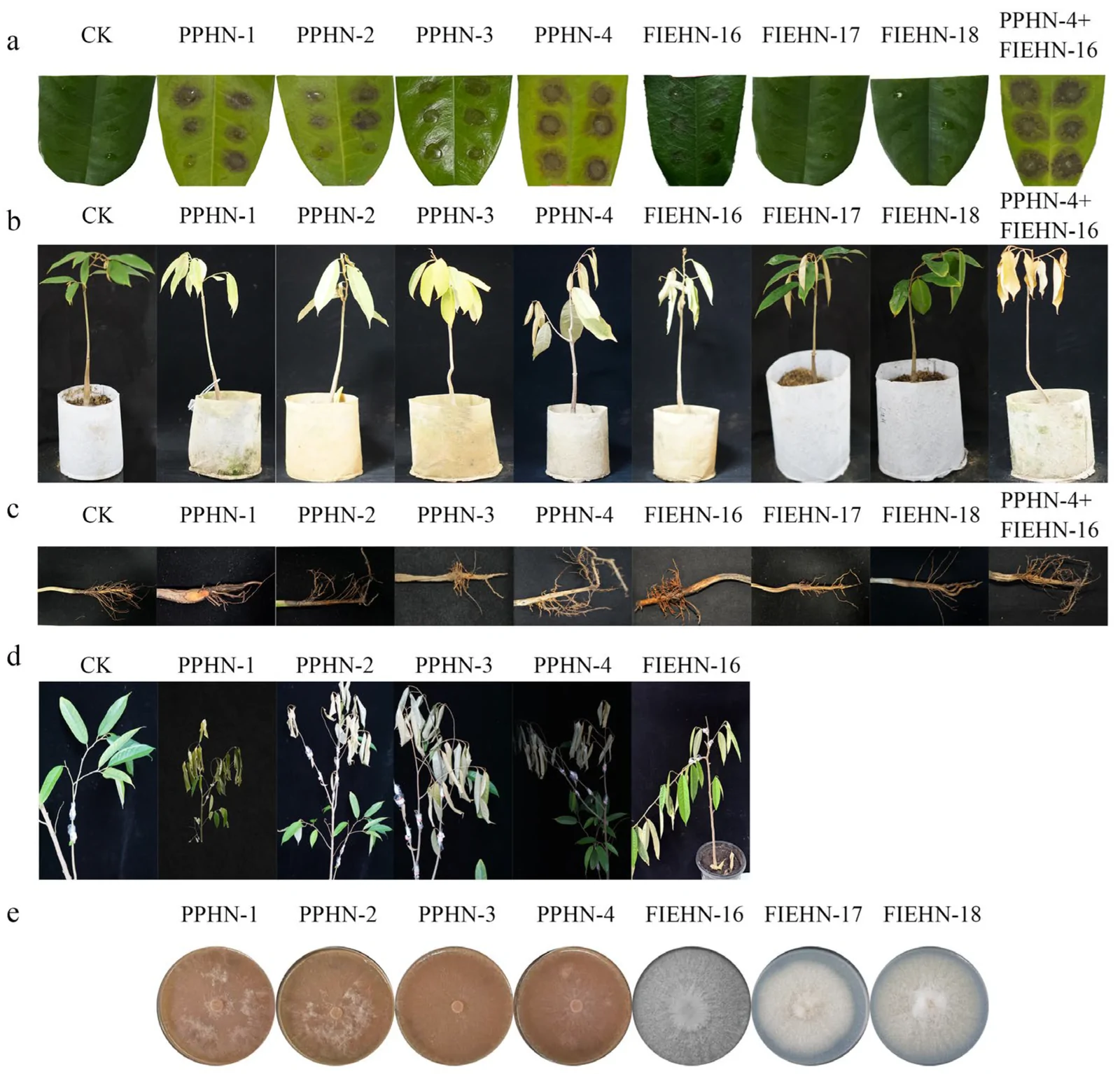
Pathogenicity assessment and fulfillment of Koch’s postulates for putative causal agents of DRR. (**a**) Detached leaf inoculation assay. Lesions induced by spore suspension inoculation with different isolates. (**b**) Root drenching assay on potted seedlings. Whole-plant symptoms at 4 weeks post-inoculation, showing leaf chlorosis, wilting, and dieback. (**c**) Root and stem base symptoms following root drenching, showing root decay, discoloration, and constriction lesions at the root–stem junction. (**d**) Stem inoculation assay. Stem canker and cortical necrosis resulting from mycelial plug inoculation. (**e**) Pathogen re-isolation. Colony morphology of isolates recovered from symptomatic tissues, identical to that of the original inoculants. Note: CK, blank control; PPHN-1 to PPHN-4, four *P. palmivora* isolates; FIEHN-16 to FIEHN-18, three FIESC isolates; PPHN-4+FIEHN-16, co-inoculation treatment.

To ascertain pathogenicity and fulfill Koch’s postulates, the suspected causative agents were administered to durian via three methods: detached leaf inoculation with spore suspensions, root drenching with spore suspensions, and stem inoculation with mycelial plugs. Substantial variation in virulence was observed among the isolates (Figure 4; Tables S4–S6). Of the four *P. palmivora* isolates tested, PPHN-4 proved the most virulent. In detached leaf assays, all PPHN isolates produced distinctive round, water-soaked lesions (Figure 4a). Root drenching with PPHN isolates rapidly induced leaf chlorosis and wilting, severe root decay, and pronounced constriction at the root-stem junction (Figure 4b,c). Stem inoculation consistently resulted in extensive cortical necrosis and canker formation (Figure 4d). By comparison, FIESC isolates were considerably less aggressive and exhibited differential host tissue adaptation: FIEHN-16 was capable of inciting leaf lesions and root symptoms but failed to infect stems, whereas FIEHN-17 and FIEHN-18 produced only mild symptoms under the test conditions.

Because field epidemics often involve polymicrobial infections, the combined effect of the highly virulent PPHN-4 and FIEHN-16 was further assessed. Co-inoculation resulted in significantly higher disease incidence and larger lesion areas on detached leaves than single-strain inoculations (Figure 4a). In root drenching assays, co-inoculation led to more severe wilting and faster symptom onset, resulting in more rapid plant mortality (Figure 4b). Although these observations suggest an interactive effect between the two pathogens, the present data do not allow a formal distinction between additive and synergistic effects, and this finding should therefore be considered preliminary. These results suggest the hypothesis that FIEHN-16, despite its limited intrinsic virulence, may facilitate *P*. *palmivora* infection and colonization, potentially by suppressing host defense responses.

The respective isolates were consistently recovered from all symptomatic inoculated tissues (Figure 4e). Re-isolated isolates were morphologically and molecularly identical to the original inoculants, as confirmed by multi-gene sequencing, whereas no target pathogens were isolated from water-treated controls, thereby fulfilling Koch’s postulates. Collectively, these data establish *P*. *palmivora* as the primary causal agent of DRR in Hainan, China, with specific FIESC isolates (notably FIEHN-16) acting as secondary contributors to disease development and epidemic progression.

Notably, among the 21 diseased samples collected, *P*. *palmivora* and FIESC were frequently co-isolated from the same individual sample—that is, from the root or stem base tissues of the same durian tree. This co-occurrence within individual host plants strongly suggests that these two pathogens can naturally co-colonize durian roots under field conditions. To determine whether such co-infection enhances disease severity, artificial co-inoculation experiments were performed using three complementary methods: detached leaf inoculation, root drenching, and stem inoculation. Across all three methods, co-inoculation significantly shortened the latent period and increased disease severity compared to single-pathogen inoculations. The field co-isolation data combined with the greenhouse co-inoculation results provide preliminary evidence that *P*. *palmivora* and FIESC can co-occur and interact on durian and that such co-inoculation may exacerbate disease severity. However, the exact nature of this interaction and its contribution to field epidemics remain to be further investigated.

### 3.3. Fungicide Sensitivity Testing

**Figure 5:**
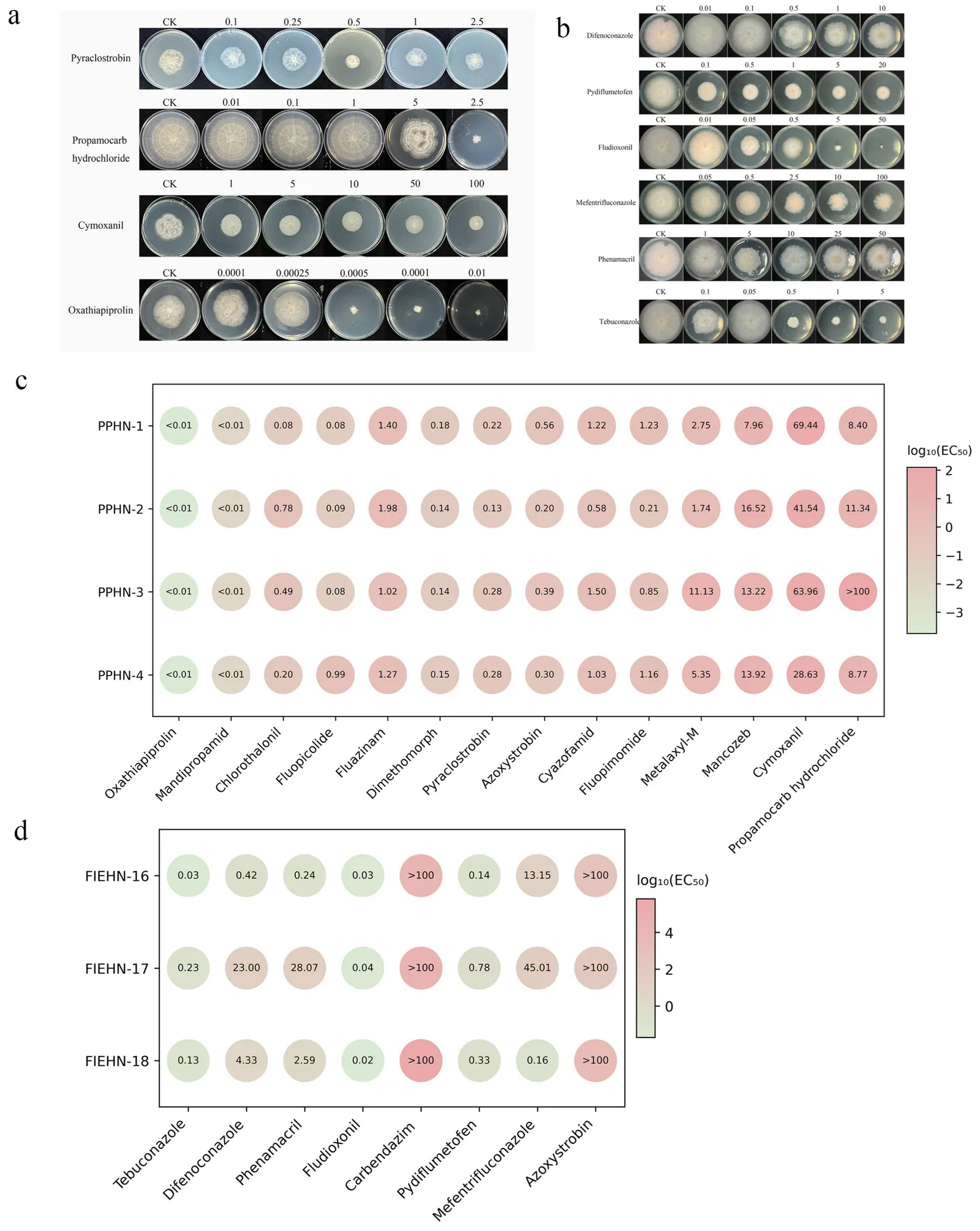
In vitro toxicity of selected fungicides against the causal agents of DRR. (**a**) Inhibitory effects of different fungicide concentrations on the mycelial growth of *P*. *palmivora*. (**b**) Inhibitory effects of different fungicide concentrations on the mycelial growth of FIESC. (**c**) Heatmap analysis of EC_50_ values for 14 fungicides against four *P*. *palmivora* isolates (PPHN 1–4). The color scale denotes log_10_-transformed EC_50_ values; green indicates high sensitivity (low EC_50_), and red indicates low sensitivity (high EC_50_). (**d**) Heatmap analysis of EC_50_ values for eight fungicides against three FIESC isolates (FIEHN 16–18). Note: all values are shown in µg/mL; CK, blank control without fungicide.

The fungicides tested in this study exhibited varying degrees of inhibition on the mycelial growth of the isolated pathogens. For the dominant oomycete pathogen *P*. *palmivora*, the sensitivity to 14 fungicides was determined. The results showed that *P*. *palmivora* was highly sensitive to multiple fungicides. Specifically, oxathiapiprolin, mandipropamid, fluopicolide, chlorothalonil, dimethomorph, azoxystrobin, pyraclostrobin, fluopimomide, cyazofamid, and fluazinam all exhibited excellent inhibitory activity, with EC_50_ value ranges of 0.000018–0.00028, 0.012–0.013, 0.08–0.99, 0.08–0.78, 0.14–0.18, 0.20–0.56, 0.13–0.28, 0.21–1.23, 0.58–1.50, and 1.02–1.98 µg/mL, respectively. In contrast, metalaxyl-M, mancozeb, cymoxanil, and propamocarb hydrochloride showed relatively low inhibitory activity, with EC_50_ value ranges of 1.74–11.13, 7.96–16.52, 28.63–69.44, and 8.40–126.58 µg/mL, respectively (Figure 5a,c). Although metalaxyl-M and mancozeb are widely used in Southeast Asia for controlling DRR caused by *P*. *palmivora*, the relatively high EC_50_ values for metalaxyl-M observed in this study may suggest reduced sensitivity.

For the synergistic fungal pathogen *Fusarium* spp. (isolates FIEHN-16, FIEHN-17, and FIEHN-18), the sensitivity to eight fungicides was evaluated: tebuconazole, difenoconazole, phenamacril, fludioxonil, carbendazim, pydiflumetofen, mefentrifluconazole, and azoxystrobin. Among the tested fungicides, fludioxonil, tebuconazole, and pydiflumetofen showed the highest efficacy against *Fusarium* spp., with EC_50_ value ranges of 0.0199–0.0362, 0.1329–0.2254, and 0.145–0.7849 µg/mL, respectively. Difenoconazole, mefentrifluconazole, and phenamacril exhibited moderate to low inhibitory activity, with EC_50_ value ranges of 0.4228–22.9961, 0.1631–45.0073, and 0.2411–28.0739 µg/mL, respectively. Because the three *Fusarium* isolates exhibited extremely high resistance levels to carbendazim and azoxystrobin, accurate EC_50_ values could not be calculated from standard dose–response curves. Therefore, resistance was assessed by determining the percent inhibition at a high discriminatory concentration of 100 µg/mL, which is more than 100-fold higher than the established sensitive baselines of 0.55 µg/mL and 0.822 µg/mL [46,47]. At this concentration, the isolates showed severe resistance: carbendazim inhibited growth by only 65.65–87.03%, and azoxystrobin by only 17.36–44.69%. Such extremely low inhibition at this high concentration clearly indicates high-level resistance to both fungicides, rendering these isolates practically insensitive to conventional field doses (Figure 5b,d). Critically, the sensitivity to azoxystrobin differed markedly between the two pathogen groups: azoxystrobin remained highly effective against *P*. *palmivora* (EC_50_: 0.20–0.56 µg/mL), whereas the FIESC isolates displayed extreme resistance, with growth virtually unaffected even at the 100 µg/mL discriminatory concentration.

### 3.4. Co-Toxicity and Greenhouse Efficacy of Fungicide Combinations Against P. palmivora Alone

**Figure 6:**
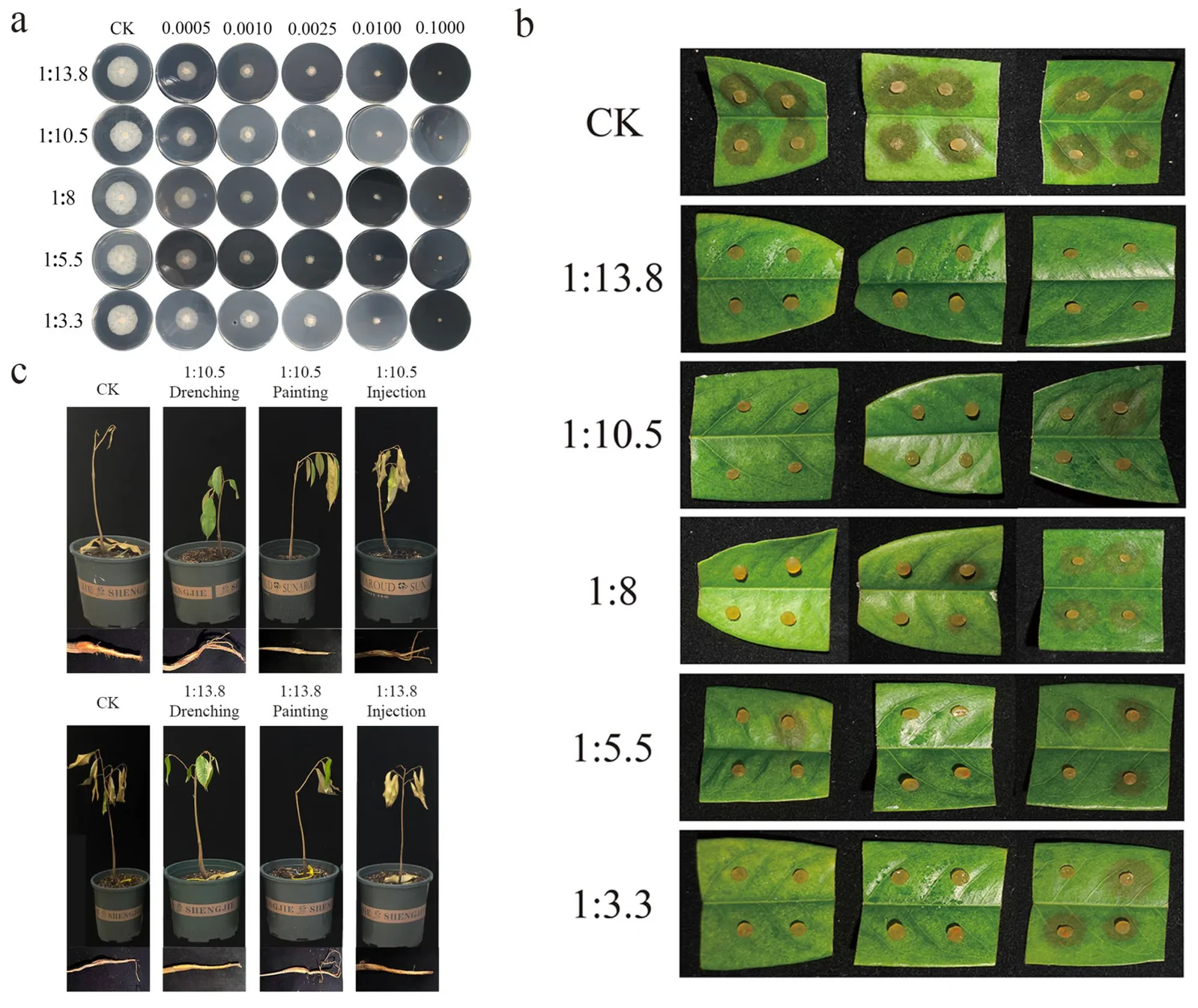
Co-toxicity and efficacy evaluation of fungicide combinations against *P*. *palmivora*. (**a**) Inhibitory effects of azoxystrobin-oxathiapiprolin mixtures at varying concentrations on the mycelial growth of *P*. *palmivora*. (**b**) Detached leaf protective assay: lesion development following treatment with different mixture ratios. The 1:13.8 and 1:10.5 ratios achieved 100% and 91.66% control efficacy, respectively. (**c**) Greenhouse efficacy assay on potted plants: comparison of three application methods (drenching, painting, and injection) at different mixture ratios. CK, water control.

To address the resistance risk of *P*. *palmivora* to commonly used compounds and to improve oomycete-specific control, the combination of oxathiapiprolin and azoxystrobin was evaluated against *P*. *palmivora* alone in both in vitro and detached leaf assays, followed by greenhouse application trials using single-pathogen inoculation. The results revealed a pronounced synergistic interaction. When oxathiapiprolin and azoxystrobin were mixed at ratios of 1:13.8 and 1:10.5, the inhibitory activity against *P*. *palmivora* mycelial growth was strongest, with synergistic ratios (SRs) of 3.57 and 2.91, respectively, both well above the synergism threshold of 1.5 (Figure 6a, Table 1). Subsequent detached leaf assays confirmed that these two ratios significantly suppressed lesion expansion, achieving protective efficacies of 100% and 91.66%, respectively (Figure 6b, Table 1).

Based on the in vitro and detached leaf results, application methods were further compared on potted plants under greenhouse conditions (Figure 6c, Table 1). The results demonstrated that root drenching was the most effective delivery method. The drenching treatment at the 1:13.8 ratio achieved the highest control efficacy of 88.89%, corresponding to a disease index of only 11.11, followed by drenching at the 1:10.5 ratio with 66.67% efficacy. Trunk painting was less effective, yielding efficacies of 66.67% and 44.44% for the two ratios, respectively. Trunk injection performed the poorest, with only 22.22% efficacy (disease index = 77.78) regardless of the ratio. In summary, the azoxystrobin-oxathiapiprolin combination at a 1:13.8 ratio applied via root drenching represents a promising strategy under the greenhouse conditions tested for managing DRR caused by *P*. *palmivora*.

### 3.5. Greenhouse Screening of Fungicide Combinations Against Mixed Inoculation of P. palmivora and FIESC

Because field samples occasionally contained both pathogens and co-inoculation increased disease severity, it was evaluated whether a combination of an anti-oomycete and an antifungal fungicide could provide broader control than single-pathogen-targeted treatments under mixed inoculation conditions. This study therefore aimed to establish a combination strategy capable of simultaneously targeting both pathogen groups. Two fungal fungicides (tebuconazole and fludioxonil) and four oomycete fungicides (oxathiapiprolin, mandipropamid, fluopicolide, and azoxystrobin) selected from earlier assays were paired in all eight possible combinations: T1, tebuconazole + oxathiapiprolin; T2, tebuconazole + mandipropamid; T3, tebuconazole + fluopicolide; T4, tebuconazole + azoxystrobin; T5, fludioxonil + oxathiapiprolin; T6, fludioxonil + mandipropamid; T7, fludioxonil + fluopicolide; T8, fludioxonil + azoxystrobin. The greenhouse efficacy of each combination against co-infection was compared to identify the optimal mixture.

**Figure 7:**
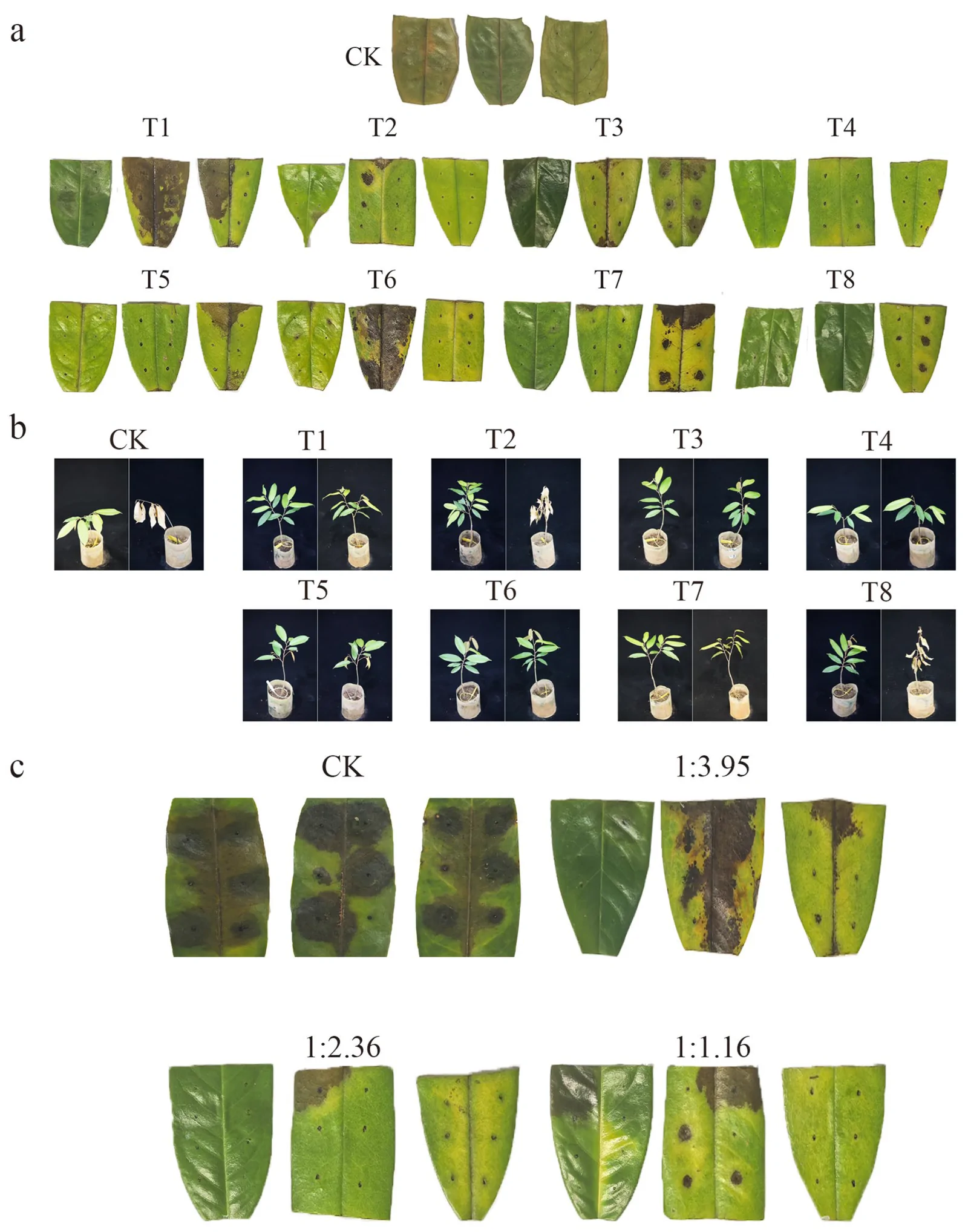
Greenhouse efficacy of fungicide combinations against co-infection in durian seedlings. (**a**) Disease symptoms on detached leaves. CK, sterile water control. (**b**) Whole-plant growth status and disease development in potted plants. T4 (tebuconazole + azoxystrobin) provided the most effective disease suppression. (**c**) Protective efficacy of tebuconazole–azoxystrobin mixtures at different ratios (1:3.95, 1:2.36, and 1:1.16) on detached durian leaves. CK, sterile water control. The 1:2.36 ratio provided the highest control efficacy (94.65%), corresponding to Table 3.

Among the eight combinations tested, the mixture of tebuconazole and azoxystrobin (T4) exhibited superior broad-spectrum efficacy (Table 2, Figure 7a,b). Specifically, T4 reduced leaf disease incidence to the lowest level (0/18) and maintained a plant disease index of only 0.70, corresponding to a control efficacy of 98.95%. Overall, this combination significantly outperformed the other treatments. In contrast, T2 (tebuconazole + mandipropamid) and T8 (fludioxonil + azoxystrobin) showed poor plant control efficacies of only 11.11% and 5.56%, respectively. Further optimization of the tebuconazole-to-azoxystrobin ratio in the T4 combination indicated that a 1:2.36 mixture provided the best dual inhibition of FIESC and *P*. *palmivora* (Figure 7c, Table 3), highlighting the considerable potential of this combination for integrated disease management under complex field conditions.

## 4. Discussion

In this study, sampling was conducted in Sanya, Baoting, and Ledong, the three principal durian-growing areas of China, which are suitable for durian grown south of 15°N and collectively account for approximately 80% of the total durian cultivation area in China. The durian industry in Hainan is still in its early developmental stage and large-scale commercial cultivation has not yet seen DRR become widespread despite recent commercial cultivation. Consequently, only 21 diseased samples with typical symptoms were obtained during the survey period. Although the sample size is relatively limited, the sampling locations cover the core durian production zones of China, and the data generated provide a preliminary but informative characterization of the pathogen composition associated with DRR in China.

DRR is widely recognized as a soilborne disease driven by complex pathogen assemblages. Through systematic field surveys and multi-locus phylogenetic analysis, this study confirmed that *P. palmivora* is the predominant causal agent of the disease in Hainan Province, China, with FIESC acting as secondary pathogens. Notably, a pronounced interaction was observed between these two pathogens, which likely constitutes a key driver of severe disease outbreaks in the field [48]. Such oomycete-fungus co-infection syndromes are not uncommon in plant pathology. For instance, in Europe, co-infection of *Pinus radiata* by *Fusarium circinatum* and *P*. *cambivora* results in exacerbated disease severity [49]. In soybean, *P*. *sojae* and *Fusarium* spp. co-infection has been shown to facilitate *P*. *sojae* infection through the action of Fpp1, a zinc alcohol dehydrogenase that catalyzes the biosynthesis of vitamin B6 (VB6), thereby compromising soybean resistance to *P*. *sojae* [24]. Additionally, the warm and humid climate of Southern China is particularly conducive to oomycete proliferation [50,51], enabling *P*. *palmivora* to dominate the pathogen community [52] and establish itself as the primary pathogen responsible for DRR.

The in vitro sensitivity assays demonstrated the fungicide’s specific action on various pathogen groups, confirming the screening and combinatorial strategy employed in this research. Against *P*. *palmivora*, the dominant pathogen, oomycete-specific fungicides such as oxathiapiprolin and azoxystrobin displayed exceptionally high inhibitory activity. Their potency is attributable to distinctive modes of action: oxathiapiprolin, a novel oxysterol-binding protein (OSBP) inhibitor, compromises cell membrane integrity by disrupting lipid metabolism [53], whereas azoxystrobin, a QoI fungicide, binds to the Qo site of cytochrome b (encoded by *cytb*) to block electron transport in the mitochondrial respiratory chain, thereby suppressing energy production [54]. In contrast, against FIESC, the conventional fungicides tebuconazole and fludioxonil were particularly effective. Tebuconazole impairs membrane permeability by inhibiting ergosterol biosynthesis [55], and fludioxonil restricts mycelial growth and interferes with glucose phosphorylation, consequently disrupting cellular metabolism. These marked differences in fungicide specificity underscore that no single active ingredient can effectively control both pathogen groups simultaneously. Given the recurring presence of co-infections between oomycete fungi and other pathogens, a single active-ingredient treatment is frequently insufficient. This study, therefore, strategically screened binary fungicide combinations targeting both pathogen groups simultaneously, representing a novel approach for managing oomycete-fungus co-infections. The tebuconazole-azoxystrobin combination delivered excellent overall control against both pathogens (96.3%). Notably, among the oomycete-targeting mixtures, specific ratios of azoxystrobin and oxathiapiprolin showed marked synergism (SR > 3.5). This synergistic effect is primarily driven by the complementarity of their target sites [56]: azoxystrobin and oxathiapiprolin simultaneously attack the energy metabolism and cell membrane biosynthetic pathways, respectively, achieving multi-site action that not only substantially improves efficacy but also reduces the risk of resistance development [57]. This combined strategy of utilizing anti-oomycete compounds offers a sensible framework for addressing intricate co-infections beyond conventional disease reduction and resistance treatments.

The choice of application method is a decisive factor in the success of tree disease management. In the present study, several delivery approaches were compared, and root drenching was identified as the most effective strategy for managing DRR. Common tree disease control methods include foliar spraying, trunk injection, and soil-directed treatments such as root drenching or broadcast application. Foliar spraying, while operationally convenient, is poorly suited for soilborne diseases because the active ingredient must penetrate the soil to reach the root–stem interface, the primary infection court, and is particularly susceptible to wash-off by the frequent rainfall typical of Southern China [58]. Trunk injection enhances the systemic distribution of the fungicide within the plant, but it causes mechanical wounding and incurs higher operational costs [59]. By contrast, root drenching delivers the fungicide directly to the target zone—the root–stem interface—thereby maximizing local concentration, markedly improving application efficiency, and minimizing environmentally driven losses [60]. The advantages of root drenching observed in this study are not limited to durian. Many devastating soilborne diseases of woody perennials, such as root and collar rot caused by *P*. *cinnamomi* in avocado, chestnut, and macadamia, similarly involve infection at the root–crown interface and could potentially benefit from this targeted application strategy [61]. The findings presented here, therefore, not only provide a practical solution for DRR management but also offer a methodological framework for optimizing fungicide delivery against a broad spectrum of soilborne diseases affecting perennial tree crops. Despite the substantial progress made in pathogen identification, fungicide screening, and delivery optimization, several limitations warrant consideration in future studies. First, although greenhouse efficacy tests can demonstrate fungicide performance, they cannot fully reproduce the complex microenvironmental conditions encountered in the field (e.g., soil pH, rainfall intensity, and diverse soil microbial communities), which may result in discrepancies between greenhouse and field-level efficacy. Second, the mixture ratios evaluated herein were derived from in vitro sensitivity tests and greenhouse co-selection and have not been validated in large-scale field trials across different climatic regimes and durian tree ages; additionally, the mixtures have yet to be tested in combination with adjuvants. Third, the potential ecological impacts of the selected fungicides on non-target organisms—such as beneficial soil microbiota and pollinators—were not assessed, though they represent an important consideration for the development of environmentally sound disease management strategies. Finally, although the azoxystrobin-tebuconazole combination exhibited excellent efficacy, its prolonged use may accelerate the evolution of QoI resistance; therefore, establishing a dynamic resistance monitoring program is essential for an early risk warning.

In summary, this study provides the first systematic characterization of the pathogen complex associated with DRR in China and identifies promising fungicide combinations and application methods under controlled greenhouse conditions. However, the efficacy data reported here are preliminary and have not been validated in large-scale field trials under different climatic conditions and tree ages. Therefore, the “tebuconazole-azoxystrobin (1:2.36)” and “oxathiapiprolin + azoxystrobin (1:13.8)” mixtures should be regarded as candidates for further field evaluation. Future studies should include multi-site field trials, resistance monitoring, and assessment of non-target ecological impacts before any practical recommendations can be made.

## 5. Conclusions

This study provides the first systematic characterization of the pathogen complex associated with durian root rot in China, identifying *P*. *palmivora* and FIESC as the predominant causal agents. Under controlled co-inoculation conditions, disease severity was higher than in single-pathogen inoculations. Critically, this study reports for the first time that these two pathogens can naturally co-infect durian and that co-infection significantly shortens the latent period and increases disease severity. Given the fundamental differences in cell wall composition and molecular targets between oomycetes and true fungi, a preliminary two-tiered chemical management system was developed: (1) a synergistic combination of oxathiapiprolin and azoxystrobin for highly effective *P*. *palmivora* suppression; and (2) a dual-target tebuconazole-azoxystrobin mixture achieving 98.95% greenhouse control efficacy against co-infection. Root drenching was identified as the optimal delivery method, precisely targeting the root–stem interface where infection initiates. The integrated framework established in this study encompasses pathogen diagnosis, fungicide resistance profiling, mixture optimization, and precision delivery. These results provide a foundation for further field validation and for the development of integrated management strategies for DRR in China and across Southeast Asian durian-producing countries. Moreover, this approach offers a valuable reference for managing *P*. *palmivora*-caused diseases on other tropical crops, including cocoa, rubber, coconut, and cassava.

## Figures and Tables

**Table 1:** In vitro toxicity and greenhouse control efficacy of oxathiapiprolin and azoxystrobin mixtures against *P*. *palmivora*.

Volume Ratio (Oxa: Azo)	EC_50_ (μg/mL)	CTC	Evaluation by CTC	SR	Evaluation by SR	Leaf Incidence (%)	Leaf Efficacy (%)	Treatment Method	Plant DI	Plant Efficacy (%)
CK	/	/	/	/	/	100	/	/	100	/
1:13.8	0.0005818	357.98	Synergism	3.5798	Synergism	0	100	Drenching	11.11	88.89
								Painting	33.33	66.67
								Injection	77.78	22.22
1:10.5	0.000667	291.08	Synergism	2.9108	Synergism	8.34	91.66	Drenching	33.33	66.67
								Painting	55.56	44.44
								Injection	77.78	22.22
1:8	0.0007983	224.25	Synergism	2.2425	Synergism	33.34	66.67	/	/	/
1:5.5	0.0007733	203.47	Synergism	2.0347	Synergism	25	75	/	/	/
1:3.3	0.0009833	130.82	Synergism	1.3082	Synergism	16.77	83.34	/	/	/

**Table 2:** Greenhouse control efficacy of different fungicide combinations against DRR caused by co-infection.

Fungicide Combination	Leaf Incidence (%)	Leaf Efficacy (%)	Plant DI	Plant Efficacy (%)
CK	100	/	66.67	/
T1	55.56	44.44	2.08	96.88
T2	11.11	88.89	59.26	11.11
T3	33.33	66.67	1.04	98.44
T4	0	100	0.7	98.95
T5	11.11	88.89	0.58	99.13
T6	27.78	72.22	0.7	98.95
T7	33.33	66.67	0.7	98.95
T8	16.67	83.33	62.96	5.56

Note: CK: Control check; T1–T8 represent different combinations of fungal and oomycete fungicides. T4 represents the mixture of tebuconazole and azoxystrobin.

**Table 3:** Protective efficacy of tebuconazole-azoxystrobin mixtures (0.1 L) at different ratios on detached durian leaves.

Mixture Ratio	Lesion Area (cm^2^)	Relative Lesion Area (%)	Control Efficacy (%)
CK	47.88 ± 6.19	59.20 ± 7.83	/
1:3.95	13.63 ± 14.62	19.33 ± 18.66	67.35
1:2.36	2.48 ± 4.30	3.17 ± 5.48	94.65
1:1.16	9.22 ± 7.99	12.39 ± 11.34	79.07

## Data Availability

The original contributions presented in this study are included in the article/Supplementary Material. Further inquiries can be directed to the corresponding author.

## Supplementary Materials

The following supporting information can be downloaded at: https://www.mdpi.com/article/10.3390/microorganisms14092061/s1, Table S1: The primers used in this study; Table S2: Concentrations of different fungicides used for EC_50_ determination; Table S3: Concentrations of different fungicides used for greenhouse efficacy evaluation; Table S4: Lesion areas on detached durian leaves inoculated with different isolates; Table S5: Disease index of plants inoculated with different isolates; Table S6: Relative lesion length on branches inoculated with different isolates; Figure S1: Morphological characterization of the dominant isolates associated with DRR.

## Abbreviation

The following abbreviation is used in this manuscript:

DRR	Durian root rot
