# Supplementary material for: Characterization and Chemical Management of Phytophthora palmivora and Fusarium incarnatum-equiseti Species Complex Associated with Durian Root Rot in China
Source: Microorganisms. 2026 Sep 15;14(9):2061. doi: 10.3390/microorganisms14092061 (PMC13609522; doi:10.3390/microorganisms14092061)
Supplement: Supplementary file 1 [file microorganisms-14-02061-s001.zip › microorganisms-4522404-supplementary.pdf]

Supporting Information

Characterization and Chemical Management of *Phytophthora palmivora* and *Fusarium incarnatum-equiseti* Species Complex Associated with Durian Root Rot in China

Yun Xing [1,2], Houya Fang [1,2], Yijia Deng [1,2], Changsheng Lu [1,3], Taorui Wu [1,2], Ting Wei [1,4], Shilong Chen [1,2], Hongna Wang [1,2], Zhiwen Wang [1,2,] *and Xili Liu [2,4]*

1 Sanya Institute, China Agricultural University, Sanya 572000, China

2 State Key Laboratory of Agricultural and Forestry Biosecurity, Ministry of Agriculture and Rural Affairs (MARA), Key Laboratory of Pest Monitoring and Green Management, College of Plant Protection, China Agricultural University, Beijing 100193, China

3 State Key Laboratory of Agricultural and Forestry Biosecurity, Key Laboratory of Biopesticide and Chemical Biology, Ministry of Education, College of Plant Protection, Fujian Agriculture and Forestry University, Fuzhou 350002, China

4 State Key Laboratory of Crop Stress Resistance and High-Efficiency Production, College of Plant Protection, Northwest A&F University, Yangling 712100, China

Table S1. The primers used in this study.

	Gene/DNA region	Primer name	Sequence (5′ → 3′)
Fusarium spp.	ITS	ITS1	TCCGTAGGTGAACCTGCGG
		ITS4	TCCTCCGCTTATTGATATGC
	EF-1α	EF-1	ATGGGTAAGGARGACAAGAC
		EF-2	GGAGGTACCAGTSATCATGTT
	rpb2	RPB2-5F2	GGGGWGAYCAGAAGAAGGC
		RPB2-7cR	CCCATRGCTTGYTTRCCCAT
Phytophthora spp.	ITS	ITS1	TCCGTAGGTGAACCTGCGG
		ITS4	TCCTCCGCTTATTGATATGC
	β-Tubulin	TUBUR1	CCTGGTACTGCTGGTACTCAG
		TUBUF2	CGGTAACAACTGGGCCAAGG
	EF-1α	ELONGF1	TCACGATCGACATTGCCCTG
		ELONGR1	ACGGCTCGAGGATGACCATG

Table S2. Concentrations of different fungicides used for EC_{50} determination.

	Fungicides	Concentration (μg/mL)	Fungicides Source
Phytophthora spp.	Metalaxyl-M	100, 25, 5, 1, 0.5, 0.25, 0.05	Sichuan Heben Crop Protection Co., Ltd.
	Azoxystrobin	25, 10, 2.5, 1, 0.5, 0.25, 0.1	Bayer AG
	Pyraclostrobin	25, 10, 2.5, 1, 0.5, 0.25, 0.1	BASF SE
	Dimethomorph	50, 10, 2.5, 1, 0.5	Jiangsu Xinyi Agrochemical Co., Ltd.
	Chlorothalonil	50, 10, 5, 2.5, 2, 1.5, 1, 0.5, 0.1	Shandong Weifang Runfeng Chemical Co., Ltd.
	Mancozeb	20, 15, 5, 2.5, 1, 0.5	Limin Chemical Co., Ltd.
	Fluopicolide	0.5, 0.25, 0.2, 0.15, 0.1, 0.05, 0.01	Jiangsu Youjia Plant Protection Co., Ltd.
	Mandipropamid	0.04, 0.02, 0.01, 0.005, 0.001	Syngenta AG
	Fluopimomide	10, 3.5, 2.5, 2, 1.5, 1, 0.5, 0.1, 0.05	Syngenta Crop Protection (Switzerland) Ltd.
	Cyazofamid	50, 25, 15, 10, 5, 2.5, 0.5, 0.1	Shandong Weifang Shuangxing Pesticide Co., Ltd.
	Fluazinam	50, 10, 2.5, 1, 0.25	Qingdao Hengning Biotechnology Co., Ltd.
	Cymoxanil	100, 50, 10, 5, 1	Shandong United Pesticide Industry Co., Ltd.
	Oxathiapiprolin	0.01, 0.001, 0.0005, 0.00025, 0.0001	Shanghai Macklin Biochemical Technology Co., Ltd.
	Propamocarb hydrochloride	1000, 200, 100, 25, 5, 1, 0.1, 0.01	Bayer AG (SL)
Fusarium spp.	Tebuconazole	5, 1, 0.5, 0.1, 0.05	Jiangsu Fengdeng Agrochemical Co., Ltd.
	Phenamacril	50, 25, 10, 5, 1	Zhejiang University
	Difenoconazole	10, 1, 0.5, 0.1, 0.01	Jiangsu Yunong Chemical Co., Ltd.
	Pydiflumetofen	20, 5, 1, 0.5, 0.1	Syngenta AG
	Mefentrifluconazole	100, 10, 2.5, 0.5,0.05	BASF SE
	Fludioxonil	50, 10, 5, 0.5, 0.05	Syngenta AG
Others	DMSO	/	Beijing Coolaber Technology Co., Ltd.
	SHAM	50	Beijing Coolaber Technology Co., Ltd.

Table S3. Concentrations of different fungicides used for greenhouse efficacy evaluation.

Fungicide Combination	Fungicide	Concentration (μg/mL)	Fungicide	Concentration (μg/mL)
1 : 13.8 (Oxa:Azo)	oxathiapiprolin	36.8	azoxystrobin	500
1 : 10.5 (Oxa:Azo)	oxathiapiprolin	45.7	azoxystrobin	480
T1	tebuconazole	100	oxathiapiprolin	20
T2	tebuconazole	100	mandipropamid	100
T3	tebuconazole	100	fluopicolide	80
T4	tebuconazole	100	azoxystrobin	250
T5	fludioxonil	330	oxathiapiprolin	20
T6	fludioxonil	330	mandipropamid	100
T7	fludioxonil	330	fluopicolide	80
T8	fludioxonil	330	azoxystrobin	250

Table S4. Lesion areas on detached durian leaves inoculated with different isolates.

Isolate	Lesion area (cm^2)
PPHN-1	14.47±2.82 c
PPHN-2	15.28±5.47 c
PPHN-3	23.32±4.04 ab
PPHN-4	21.49±2.05 b
FIEHN-16	2.83±2.56 d
FIEHN-17	0.13±0.11 d
FIEHN-18	0
PPHN-4+FIEHN-16	24.85±1.50 a

Table S5. Disease index of plants inoculated with different isolates.

Isolate	Latent period (days)	DI
PPHN-1	8	55.6
PPHN-2	12	25.9
PPHN-3	9	48.1
PPHN-4	15	70.4
FIEHN-16	20	18.5
FIEHN-17	25	11.1
FIEHN-18	30	11.1
PPHN-4 + FIEHN-16	12	70.4

Note: Data represent the mean Disease Index (DI) of three biological replicates (n=3) for each treatment group.

Table S6. Relative lesion length on branches inoculated with different isolates.

Isolate	Relative lesion length (%)
PPHN-1	51.8±0.85 b
PPHN-2	46.65±1.48 c
PPHN-3	50.6±0.71 b
PPHN-4	71.05±1.77 a
FIEHN-16	0

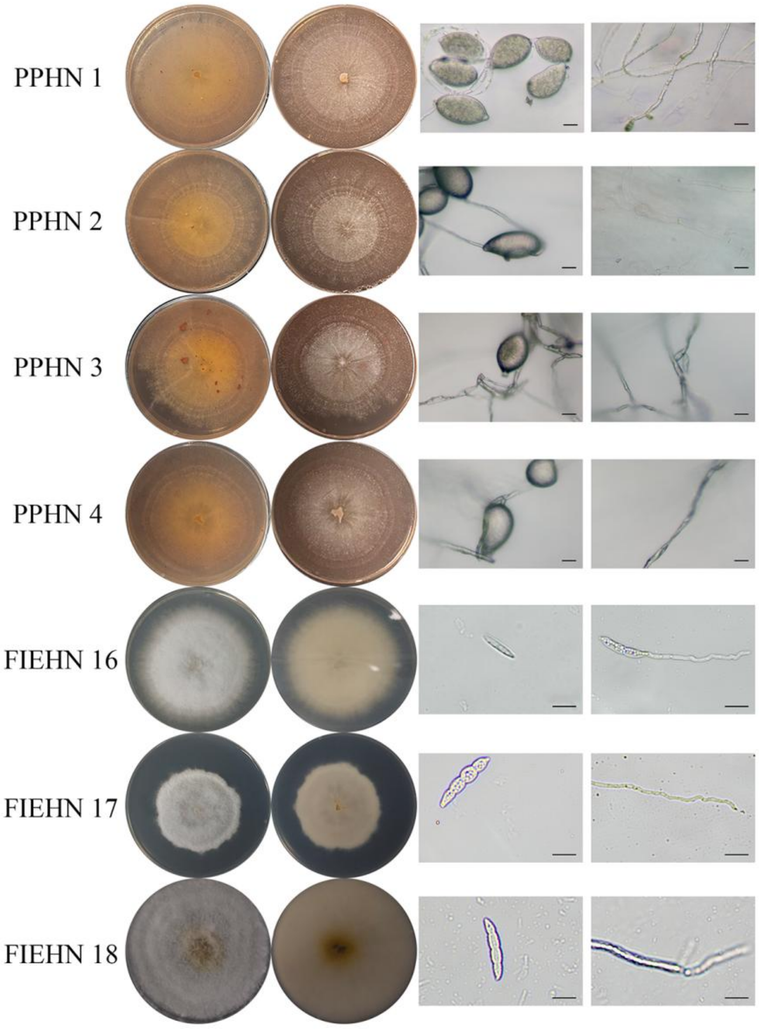

Figure S1. Morphological characterization of the dominant isolates associated with DRR.

Upper panel: *Phytophthora palmivora* (isolates PPHN 1–4). Lower panel: the *Fusarium incarnatum-equiseti* species complex (FIEHN 16 – 18), comprising all three Isolates of the Fum-3 cluster confirmed by pathogenicity assays. For each isolate, colony upper and reverse surfaces on PDA and micrographs of spores/sporangia and mycelia are shown from left to right. Scale bar = 10 µm.
